# Isorhamnetin Induces Apoptosis and Suppresses Metastasis of Human Endometrial Carcinoma Ishikawa Cells via Endoplasmic Reticulum Stress Promotion and Matrix Metalloproteinase-2/9 Inhibition In Vitro and In Vivo

**DOI:** 10.3390/foods11213415

**Published:** 2022-10-28

**Authors:** Lei Ye, Run-Hui Ma, Xiu-Xiu Zhang, Kiran Thakur, Jian-Guo Zhang, Mohammad Rizwan Khan, Rosa Busquets, Zhao-Jun Wei

**Affiliations:** 1 School of Food Science and Biological Engineering, Hefei University of Technology, Hefei 230009, China; 2 School of Biological Science and Engineering, North Minzu University, Yinchuan 750021, China; 3 Department of Chemistry, College of Science, King Saud University, Riyadh 11451, Saudi Arabia; 4 School of Life Sciences, Pharmacy and Chemistry, Kingston University London, London KT1 2EE, UK

**Keywords:** endometrial cancer, Ishikawa, isorhamnetin, UPR response, mitochondria

## Abstract

Endometrial cancer (EC) is a very common female cancer which has attracted more and more attention. According to the individual patient’s condition, the current treatment of EC patients is mainly based on surgery, which is supplemented by chemotherapy, radiotherapy, and endocrine intervention. However, these existing treatment strategies also have some inevitable limitations. Therefore, it is particularly important to find an active ingredient with low toxicity and a high safety profile against EC. Isorhamnetin is a flavonoid known to be present in a variety of plants, such as sea buckthorn, dry willow, and wolfberry. In recent years, the anti-tumor effects of isorhamnetin have been reported. In our study, isorhamnetin was shown to induce apoptosis in Ishikawa cells by inducing the endogenous mitochondrial apoptotic pathway and exogenous death receptor pathway, promoting the endoplasmic reticulum stress-related pathway, and activating the corresponding markers of UPR response. In addition, isorhamnetin affected the expression of MMP2 and MMP9-related proteins in vitro and in vivo and eventually repressed metastasis. Therefore, isorhamnetin can be used as a promising medicinal material for the treatment of EC.

## 1. Introduction

Endometrial cancer (EC) has a high incidence among female reproductive system tumors and seriously affects women’s reproductive health. It is the third most common malignancy causing death in women, behind only ovarian cancer and cervical cancer [1,2]. EC originates from the endometrium and is highly invasive and metastatic, which makes the treatment of EC patients extremely challenging [3]. Depending on the conditions of individual patients, the current treatment for EC patients is mainly surgical treatment with chemotherapy, radiotherapy, and endocrine intervention as adjuvant therapy. However, this treatment strategy has inevitable drawbacks, such as surgery possibly leading to the loss of fertility and endocrine intervention therapy possibly generating drug resistance [4]. Therefore, it is of utmost urgency to discover some novel therapeutic modalities with low toxicity and strong therapeutic targets. Currently, the most familiar EC cell lines are Ishikawa, KLE, etc. The Ishikawa cell line was first discovered by Nishida et al. [5]. Subsequent histological studies of tumors in nude mice showed it to be a well-differentiated adenocarcinoma, very similar to the original human tumor, and thus suitable for EC studies.

In recent years, antineoplastic drugs of plant origin have attracted significant attention worldwide. These plant compounds can provide unique new chemical structures for the design of more desirable novel drugs [6,7,8], which can also be lead compounds for the creation of new drugs. Among the natural compounds, isorhamnetin is a flavonoid known to be present in a variety of plants, such as sea buckthorn, dry willow, and wolfberry. Recent studies have shown that isorhamnetin and its glycoside form can exert a wide range of pharmacological effects and health benefits, including cardio-cerebrovascular benefits, nerve protection, anti-inflammation, kidney protection, lung protection, anti-osteoporosis, anti-oxidation, prevention of obesity, and anti-tumor effects [9]. The various effects of isorhamnetin play a significant role in the treatment of many kinds of diseases by medicating some signaling pathways and cytokines, presenting high medicinal value [10].

Apoptosis acts as a vital character in the fate decision process of some tumor cells [11] and undergoes morphological changes in cell contraction, which are accompanied by a reduced degree of cell–cell adhesion and DNA fragmentation response [12,13,14]. The exogenous death receptor pathway is a classical apoptotic response caused by the combination of plasma membrane death receptors with extracellular ligands. When a cell is stimulated by an external signal, the plasma membrane death receptor binds to the extracellular ligand, causing a change in the structural domain of the receptor and, in response to this change, many cytoplasmic proteins of the death structural domain can bind to it, eventually activating Caspase-8 initiation which further activates the execution precursor Caspase-3, ultimately triggering downstream programmed death [15].

In addition to death receptor exogenous signals, endogenous cellular signals such as DNA damage can also induce the onset of apoptosis. When DNA damage occurs inside the cell, it prevents efficient scavenging of large amounts of free radicals and causes accumulation of high concentrations of calcium ions inside the cell. This intracellular change can induce the activation of pro-apoptotic proteins in the Bcl-2 family, which can alter the permeability of mitochondria, thereby helping cytochrome C to be released from the mitochondrial membrane gap and enter the cytoplasmic lysis to bind Apaf-1 and the precursor Caspase-9 to form apoptotic vesicles and finally activate Caspase-3, triggering the onset of apoptosis [16].

Metastasis of tumor cells is a collection of intricate and complex processes, including cell migration, invasion, and other related processes [17]. It refers to the process by which a tumor diverges from its original location to different target tissues through various pathways. Cancer invasion and metastasis are the most difficult stages of cancer treatment; therefore, controlling cancer cell metastasis has been the most challenging part of the treatment of various cancers in recent years [18]. Epithelial–mesenchymal transition is very important and associated with cell migration and invasive growth; it reshapes the stability of the extracellular matrix [19] and contributes to the maintenance of intracellular homeostasis. E-cadherin, one of the important proteins of epithelial–mesenchymal transition, regulates adhesion connection establishment, thus forming a continuous adherent epithelium and maintaining the homeostasis of the intracellular environment. Once the above process is disrupted, it will affect cell metastasis [20]. Therefore, the treatment of cancer can be effectively inhibited by regulating the pathways associated with cancer cell metastasis.

To date, there is a limited amount of data available on the effects of isorhamnetin on mitochondrial pathways and endoplasmic reticulum stress associated with apoptosis and metastasis in human EC treatment. Therefore, we explored the effects of isorhamnetin on the growth, morphology, cell cycle, apoptosis, and metastasis of EC in vitro. Moreover, some effects of isorhamnetin on apoptosis and the metastasis of Ishikawa tumor-bearing mice were also investigated by immunohistochemistry using an in vivo EC model. It is speculated that our results can provide new approaches for the treatment of EC.

## 2. Materials and Methods

### 2.1. Materials and Chemicals

Isorhamnetin standards were purchased from Must Bio-Technology Co., Ltd. (Purity > 98%) (Chengdu, China), cell cycle and apoptosis assay kits were procured from Beyotime Biotechnology (Shanghai, China), and human endometrial carcinoma Ishikawa cells were purchased from Wuhan Pronset Life Sciences Co. (Wuhan, China). Antibodies were purchased from Abcam (Cambridge, UK) and Cell Signaling Technology (Danvers, MA, USA).

### 2.2. Cell Culture

When the cells were polygonal in shape and firmly attached, the supernatant culture medium was discarded and 1 × phosphate-buffered saline (PBS) was added and the mixture was gently shaken. After that, 1 mL of trypsin was added for cell digestion. After digestion, the reaction was terminated with 1 mL of fresh medium followed by centrifugation (1200 rpm, 5 min) [21]. After centrifugation, the supernatant was discarded and 1 mL of fresh medium was added to resuspend the cells. The resuspended cells were aspirated and slowly spotted into the culture dish by the 5-point method and then placed in an incubator.

### 2.3. Cell Inhibition Assay

Cells were incubated with different concentrations of isorhamnetin for 24 h. After MTT (3-(4,5-dimethylthiazol-2)-2,5-diphenyltetrazolium bromide salt) treatment, the absorbance values were measured at the detection wavelengths of 490 nm and 630 nm and a full-wavelength enzyme marker (TS100, Nikon, Tokyo, Japan) was used for calculation [22].

### 2.4. Tumor-Bearing Mice Assay

All the protocols of the Ishikawa cell-based tumor-bearing mice assay were performed according to the National Institute of Health’s Guide for the Care and Use of Laboratory Animals and approved by the Animal Ethics and Use Committee of the Hefei University of Technology (SYXK (Anhui) 2021—007). The 4~5-week-old mice were selected and then fed for one week to construct a tumor-bearing mouse model constructed from Ishikawa cells from EC. When the tumors grew to 90 mm^3^, 16 mice were randomly divided into two groups by average size [23]. After grouping, the isorhamnetin-treated group was injected intraperitoneally with 20 mg/kg of drug solution (isorhamnetin-soluble DMSO) once a day for 15 days. The anti-tumor effect of the solution was observed by measuring the diameter of the transplanted tumors in nude mice with vernier calipers every two days and calculating the corresponding volume by the formula 0.5 × length × (width) 2, with dietary intake and body weight changes also recorded. After 15 days of administration, all the mice were asphyxiated with CO2 and their body weights were recorded. The tumors were surgically removed and weighed, which was then followed by the fixation of different organs; tumor tissues were fixed with 4% paraformaldehyde, embedded using paraffin, and used for hematoxylin and eosin (H&E) staining, as well as immunohistochemistry and other related tests.

### 2.5. H&E Staining Test

Different organs from the control and isorhamnetin-treated nude mice group were fixed with 4% paraformaldehyde, and these tissues were transferred to embedding boxes and rinsed in running water for 20–30 min in order to remove the fixative from the tissues. Excess water was gradually removed from the tissues, using alcohol as a dehydrating agent, with a gradient elution concentration. Tissues were transparently treated with xylene, a clearing agent, before paraffin embedding, cooling, and fixation. The wax blocks were cut with a tissue slicer and subsequently dried. The dried sections were sequentially placed in different concentration gradients of xylene solution and alcoholic aqueous solution for dewaxing and washing. After the dewaxing and washing were completed, staining was performed. The sections were stained in hematoxylin for 5–8 min and rinsed with running water after staining. Subsequently, 1% hydrochloric acid and ammonia were added to separate the tissues, which were then rinsed with running water. After rinsing, the eosin staining solution was added to the tissue, which was left to stain for 2~3 min. After eosin staining, the sections were then dehydrated in aqueous alcohol and xylene [24]. Finally, the sections were removed from xylene, dried, sealed with neutral chewing gum, and observed with a microscope before three fields of view were then randomly selected for recording (80i, Nikon, Japan).

### 2.6. Cell Morphology Observation

Ishikawa cells were collected and inoculated, 2 mL was added to each well, and cells were then incubated for 24 h. If they were plastered, the medium in the six-well plate was removed by pipetting and 2 mL of medium containing different concentrations of isorhamnetin (20 μM, 40 μM, and 60 μM) was added to the group. After incubation, the medium was discarded and then washed with PBS and observed.

### 2.7. Cell Cycle Assay

Ishikawa cells were collected and inoculated (2 mL) in each well. After washing with 1 × PBS, 1 mL of chilled 70% ethanol was added into each centrifuge tube containing cells that had been kept overnight at 4 °C [25]. The cells were precipitated by centrifugation at 1000 g for 5 min and the supernatant was carefully removed. The cells were then resuspended by adding 1 mL of 1 × PBS and centrifuged again. The cells were detected by flow cytometry (Beckman Coulter, Pasadena, CA, USA) at an excitation wavelength of 488 nm, and the results were statistically analyzed using Flowjo software.

### 2.8. Apoptosis Assay

After treatment with 20, 40, and 60 μM of isorhamnetin for 24 h, walled cells and suspended cells were collected in 1.5 mL sterile EP tubes, washed with 1 × PBS, and 400 μL of Annexin V-FITC conjugate was gently added to the collected cell sediment [23]. These cells were resuspended and stained with Annexin V-FITC for 15 min (5 μL) and propidium iodide for 15 min (10 μL). After staining, the cells were detected by flow cytometry (Beckman Coulter, Pasadena, CA, USA). All results were statistically analyzed with Flowjo software.

### 2.9. Cellular Calcium Ion Permeation Assay

Ishikawa cells at the logarithmic proliferation stage were transferred (1 × 10^5^ cells/mL) to 6-well plates for the test [22]. Each group was repeated 3 times and incubated overnight in a constant temperature incubator. On the next day, the cells were observed to verify adherence and, if the cells were adherent, the medium in the six-well plate was pipetted off and 2 mL of medium containing different concentrations of isorhamnetin was added. After 24 h of treatment, the adherent and suspended cells were collected in 1.5 mL sterile EP tubes. After washing with 1 × PBS, 5 μM of Fluo-3 AM was added to different concentrations of isorhamnetin tubes before being incubated at 37 °C for 30 min. The incubation period was then reversed and cells were mixed every 5 min. The cells were resuspended with PBS and detected at an excitation wavelength of 488 nm by flow cytometry (Beckman Coulter, Pasadena, CA, USA).

### 2.10. Reactive Oxygen Assay

After collecting Ishikawa cells treated with isorhamnetin, Ishikawa cells were resuspended with DCFH-DA (0.5 μM) and incubated in an incubator at 37 °C for 20 min [26]. The cells were washed three times with a serum-free medium, and the cells were immediately examined at an excitation wavelength of 488 nm before being statistically analyzed with Flowjo software.

### 2.11. Cell Scratch Heal Test

After the cells had grown to a single cell layer after being cultured for 24 h, 200 μL was used to scratch a straight line wound on the monolayer of cells. The cell debris generated in the cell line was then removed by washing the sample three times with PBS [24]. The remaining walled cells were treated with different concentrations of isorhamnetin for 24 h. Photographs were taken at different times (0 h and 24 h) with a microscope (TS100, Nikon, Japan). Three fields of view were randomly selected from each cell plate culture well for recording. The area of the scratches in the photographed images in each well was measured by Image J software.

### 2.12. Cell Migration and Invasion Assay

A Transwell assay was used to detect the invasion and migration of Ishikawa cells with different concentrations of isorhamnetin. After collecting cells treated with different concentrations of isorhamnetin, Ishikawa cells were resuspended in serum-free DMEM cell medium until the final concentration of cells was 5 × 10^5^ cells/mL. In the Transwell assay, cell suspensions with different concentrations of isorhamnetin were cultured in the upper chamber, and 90% DMEM + 10% FBS were put in the lower chamber [27]. After isorhamnetin treatment, the upper chambers were washed with sterilized distilled water to remove the excess cell culture medium, which was followed by fixation with 4% paraformaldehyde for 10–15 min, washing with distilled water, and staining with crystal violet staining solution for 20 min. After washing with distilled water to remove excess staining solution, three fields were randomly selected from each chamber of the Ishikawa cells cultured for 24 h using a microscope (TS100, Nikon, Japan). Three randomly selected fields of view were photographed. For cell invasion experiments, before collecting the cells, the stromal gel was diluted 5 times using a serum-free medium. After the stromal gel was prepared, 200 μL was added to the upper chamber of the Transwell and solidified in the incubator at 37 °C for 30–50 min before different concentrations of isorhamnetin were added to the upper chamber. The rest of the treatment and observation procedures were the same as cell migration.

### 2.13. Cell Protein Extraction and Western Blotting Experiments

After isorhamnetin treatment, 500 μL of trypsin digestion solution was added to each well, and digestion was allowed to take place for 3–5 min. After washing, the supernatant was discarded. The cells were added into RIPA cell lysate depending on the number of cells deposited at the bottom of the centrifuge tube, which was then followed by centrifugation (15,000 rpm, 20 min). The protein concentrations of the cells in the isorhamnetin-treated group were determined according to the procedure of the BCA protein assay kit. The protein mixture was diluted by adding 5 × loading buffer in a centrifuge tube at a ratio of 1:4 and then stored in a water bath for 10 min at 98.3 °C. Western blotting included protein electrophoresis, protein transfer, and antibody hybridization; the PVDF membranes were then removed and placed in a chemiluminescence apparatus for 5 min with drop wise addition of contrast solution and photographed. The expression of the remaining target proteins was compared with the Image J software using β-actin as the internal reference protein [28].

### 2.14. Immunohistochemistry

Paraffin sections were dewaxed and rehydrated, which was then followed by false-positive removal, serum blocking, primary antibody addition, secondary antibody addition, DAB color development, nuclei re-staining, and microscopic examination (Leica, Wetzlar, Germany). Three fields of view for each sample were randomly selected for photographic recording [22].

### 2.15. Data Analysis

The experiments were repeated at least three times in vitro, and nude mice experiments had eight mouse samples per group. Data were counted and calculated using Excel 2019 (Microsoft, Redmond, WA, USA). Statistical analysis was performed using Origin 8.6 and Duncan‘s test (SPSS), which was performed to compare the treatment group and the control group. Statistical significance for all tests was expressed as mean ± standard deviation (n ≥ 3) at a significance level of *p* < 0.05.

## 3. Results and Discussion

### 3.1. Consequence of Isorhamnetin on the Proliferation of Ishikawa Cells

**Figure 1:**
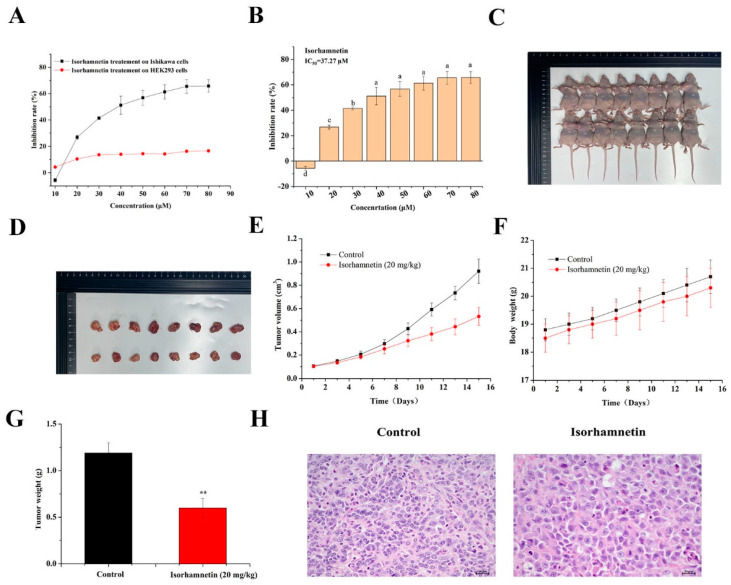
Effect of isorhamnetin on the proliferation of Ishikawa cells and Ishikawa xenograft mice. (**A**): The 24 h inhibitory effect of isorhamnetin on Ishikawa cell and HEK 293 cell proliferation. (**B**): Bar chart of the inhibitory effect of isorhamnetin on proliferation. (**C**): Effect of isorhamnetin (20 mg/kg) on Ishikawa xenograft mice (*n* = 8). (**D**): Effect of isorhamnetin (20 mg/kg) on tumor size in Ishikawa xenograft mice (*n* = 8). (**E**): A line graph of the tumor volume in control and isorhamnetin–treated groups (20 mg/kg). (**F**): Line graph of the body weight of mice in the control and isorhamnetin groups (20 mg/kg). (**G**): Tumor volume of the control and isorhamnetin–treated groups (20 mg/kg). (**H**): H&E staining of tumors in the control and isorhamnetin–treated groups (20 mg/kg). The experiments were repeated at least three times in vitro, and nude mice experiments had at least eight mouse samples per group in vivo. Different alphabetic letters (including a, b, c, and d) denote significant differences between different groups (*p* < 0.05, *n* > 3). ** *p* < 0.01 included to show group differences in the in vivo experiment.

The inhibitory effect of isorhamnetin on the proliferation of Ishikawa cells was investigated by MTT assay as shown in Figure 1A,B. The proliferation of Ishikawa cells was inhibited after 24 h of isorhamnetin treatment. The fitted curves showed that the semi-inhibitory concentration (IC50) of isorhamnetin on Ishikawa cells was 37.27 μM.

### 3.2. Effect of Isorhamnetin on the Growth of Tumors in Ishikawa Tumor-Bearing Mice

As shown in Figure 1C–E, the growth of tumors in the isorhamnetin-treated group was significantly inhibited, with the inhibition rate of the tumor as high as 50.42% (Figure 1G). The tumor cells in the isorhamnetin-treated group were rounder or fat and shuttle-shaped, the nuclei were larger and darker, and the tumor cells were mildly necrotic, as shown in H&E staining compared to the control group (Figure 1H). The tumor cells in the isorhamnetin-treated group were rounder or fat and spindle-shaped, with larger and darker colored nuclei and mild necrosis of tumor cells (Figure 1F). The above results suggest that isorhamnetin can inhibit the growth of tumor formation in Ishikawa tumor-bearing mice with EC.

### 3.3. Effect of Isorhamnetin on the Morphology of Ishikawa Cells

**Figure 2:**
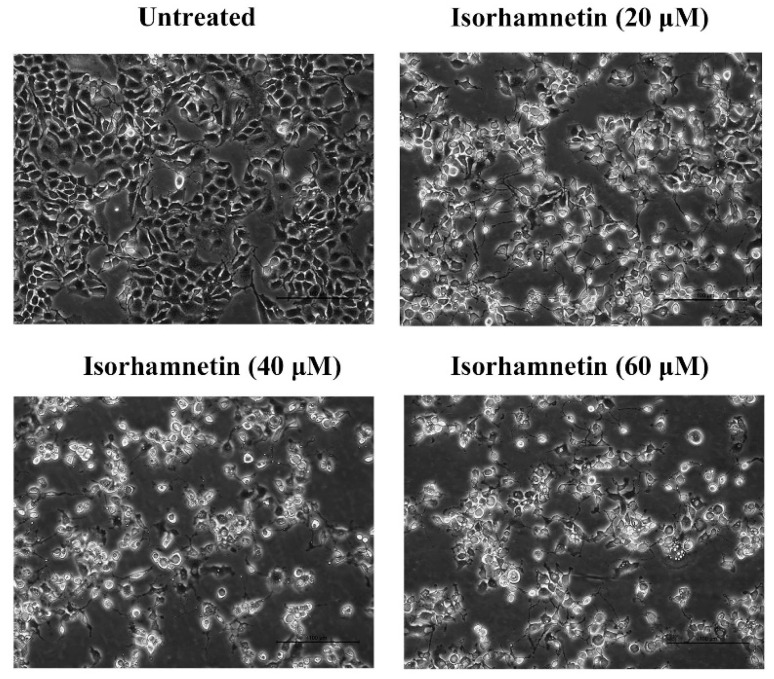
The morphology of Ishikawa cells treated with different concentrations of isorhamnetin (0 μM, 20 μM, 40 μM, and 60 μM).

The number of morphologically altered cells was increased significantly with the different concentrations of isorhamnetin, and the morphology gradually changed from the irregular shape of the untreated group to the round shape while exhibiting some vacuolation (Figure 2).

### 3.4. Isorhamnetin Blocks the G2/M Phase in Ishikawa Cells

**Figure 3:**
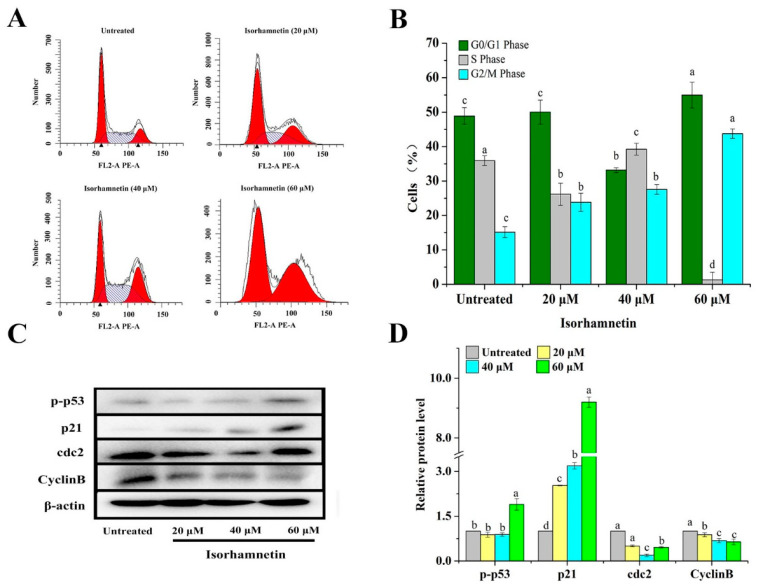
Alternation of cell cycles after isorhamnetin treatment on Ishikawa cells. (**A**): Flow diagram of the effect of different concentrations of isorhamnetin (0 μM, 20 μM, 40 μM, and 60 μM) on the Ishikawa cell cycle. (**B**): Quantification of the effects of isorhamnetin (0 μM, 20 μM, 40 μM, and 60 μM) on the Ishikawa cell cycle. (**C**): Effect of isorhamnetin on p-p53, p21, cdc2, and CyclinB in the G2/M phase of the Ishikawa cell cycle. (**D**): Quantitative protein map of the effect of isorhamnetin on p-p53, p21, cdc2, and CyclinB in the G2/M phase of the Ishikawa cell cycle. Different alphabetic letters (a, b, c, d) denote significant differences between different groups (*p* < 0.05, *n* ≥ 3).

Cell cycle disruption is one of the mechanisms by which tumor growth is inhibited. We explored the effect of isorhamnetin on the Ishikawa cell cycle and found that isorhamnetin can block the progression of the G2/M phase in Ishikawa cells, which is probably due to the activation of p53 as an upstream regulatory protein of the cell cycle and forms p-p53 in response to isorhamnetin treatment, which in turn activates its downstream protein p21 which then acts as a switch regulating cell mitosis. As a result, the threonine 14 site of cdc2 and the tryptophan 15 site of cdc2 could not be effectively phosphorylated and could not bind to the maturation promoting factor (MPF), thus hindering the cell cycle G2/M process [29]. If damaged cells are not repaired promptly, cell cycle arrest may lead to programmed cell death. In order to investigate the mechanism of action of isorhamnetin on human EC Ishikawa cells, the next step was to examine both cell proliferation and apoptosis. After co-culture with Ishikawa cells and different concentrations of isorhamnetin for 24 h, cells were blocked in the G2/M phase and increased from 15.17% to 43.76% with increasing drug concentration, while cells in the S phase decreased from 35.93% to 1.27% (Figure 3A,B). These phenomena suggest that isorhamnetin can block the cell cycle of Ishikawa cells in the G2/M phase.

In order to further investigate the mechanism of the cell cycle influenced by isorhamnetin, we investigated protein expression levels. The expression of Cyclin B and cdc2 was significantly decreased, but the expression of p-p53 and p21 upstream of them was remarkably increased (Figure 3C,D). The above results show that isorhamnetin can inhibit the proliferation of Ishikawa cells in the G2/M phase [30].

### 3.5. Isorhamnetin Promotes Apoptosis in Ishikawa Cells

**Figure 4:**
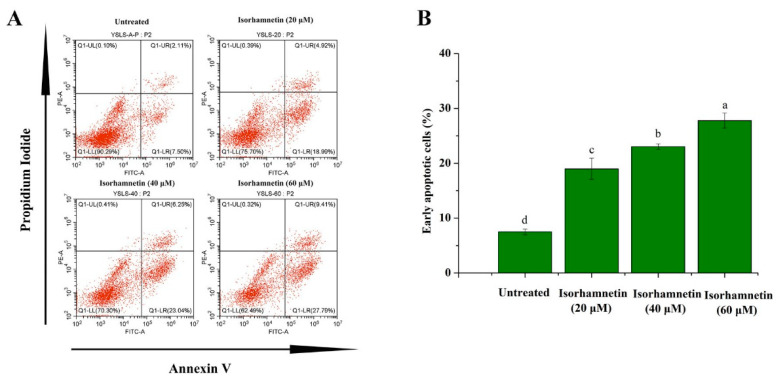
Apoptosis of Ishikawa cells treated with isorhamnetin. (**A**): Flow diagram of the effect of different concentrations of isorhamnetin (0 μM, 20 μM, 40 μM, and 60 μM) on apoptosis. (**B**): Quantitative analysis of the effects of isorhamnetin on early apoptotic cells with different concentrations of isorhamnetin (0 μM, 20 μM, 40 μM, and 60 μM). Different alphabetic letters (a, b, c, d) denote significant differences between different groups (*p* < 0.05, *n* ≥ 3).

The apoptosis rates of early apoptotic cells were 7.5% ± 0.51%, 18.99% ± 1.95%, 23.04% ± 0.15%, and 27.29% ± 1.39%, respectively (Figure 4A,B). The above results suggest that isorhamnetin can induce apoptosis in Ishikawa cells in a dose-dependent manner.

### 3.6. Effect of Isorhamnetin on Calcium Leakage from the Endoplasmic Reticulum of Ishikawa Cells

**Figure 5:**
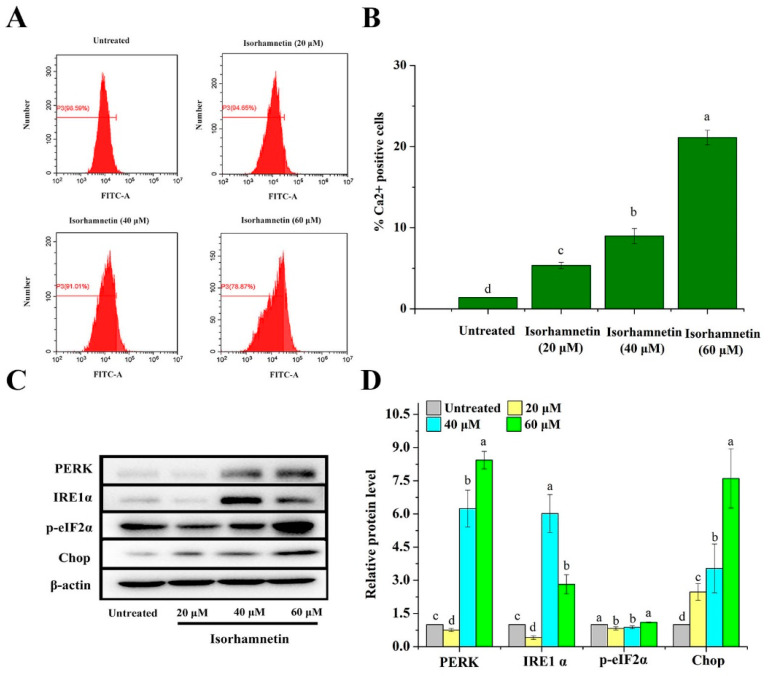
Endoplasmic reticulum stress on Ishikawa cells treated with isorhamnetin. (**A**): Flow diagram of the effect of different concentrations of isorhamnetin (0 μM, 20 μM, 40 μM, and 60 μM) on calcium ion permeation. (**B**): Quantitative analysis of the effects of isorhamnetin (0 μM, 20 μM, 40 μM, and 60 μM) on calcium ion permeation. (**C**): Effect of isorhamnetin on PERK, IRE1α, p-eIF2α, and Chop under endoplasmic reticulum stress. (**D**): Quantitative protein map of the effect of isorhamnetin on PERK, IRE1α, p-eif2α, and Chop under endoplasmic reticulum stress. Different alphabetic letters (a, b, c, d) denote significant differences between different groups (*p* < 0.05, *n* ≥ 3).

In a normal environment, the endoplasmic reticulum is responsible for the correct processing and folding of proteins; however, once the intracellular environment is disturbed, proteins fail to fold and are processed incorrectly due to a lack of molecular chaperones, cellular energy delivery, Ca^2+^ deficiency, impairment of the redox environment, protein mutation, and disulfide bond reduction [31,32,33]. After staining with Fluo-3 AM fluorescent dye [34] and observation by flow cytometry, the intracellular calcium ion permeation rates after treatment with isorhamnetin were found to be 1.41% ± 0.01%, 5.35% ± 0.0.87%, 8.99% ± 0.93%, and 21.13% ± 1.90%, respectively (Figure 5A,B). The above results suggest that isorhamnetin can affect the release of calcium ions from Ishikawa cells. Unfolded proteins were accumulated in the endoplasmic reticulum, which then leads to a UPR response under ER stress that allows the UPR sensor to dissociate from PERK, ATF6, and IRE1α and thus induces downstream apoptotic signaling. Among these proteins, PERK activates cell membrane structural domains through dimerization and autophosphorylation, which in turn phosphorylates and activates the downstream regulator eIF2α. In addition, it phosphorylates Nrf-2 activated by PERK, which in turn dissociates from Keap1 and becomes a nuclear transcription factor in the nucleus to regulate downstream protein expression. IRE1α contains an endonuclease structural domain which is activated by dimerization and autophosphorylation. Activated IRE1α induces the expression of XBP1s mRNA, which translates into an active transcription factor. Furthermore, the kinase structures of TRAF2 and ASK1 were also activated by IRE1α, which eventually induces apoptosis [35,36]. PERK and IRE1α, key regulatory proteins of endoplasmic reticulum stress, were significantly increased, which in turn promoted the expression of the Chop protein downstream (Figure 5C,D), an important transcription factor that can lead to DNA damage and thus apoptosis [37].

### 3.7. Effect of Isorhamnetin on Mitochondrial Apoptosis and Death Receptor Signaling Pathways in Ishikawa Cells

**Figure 6:**
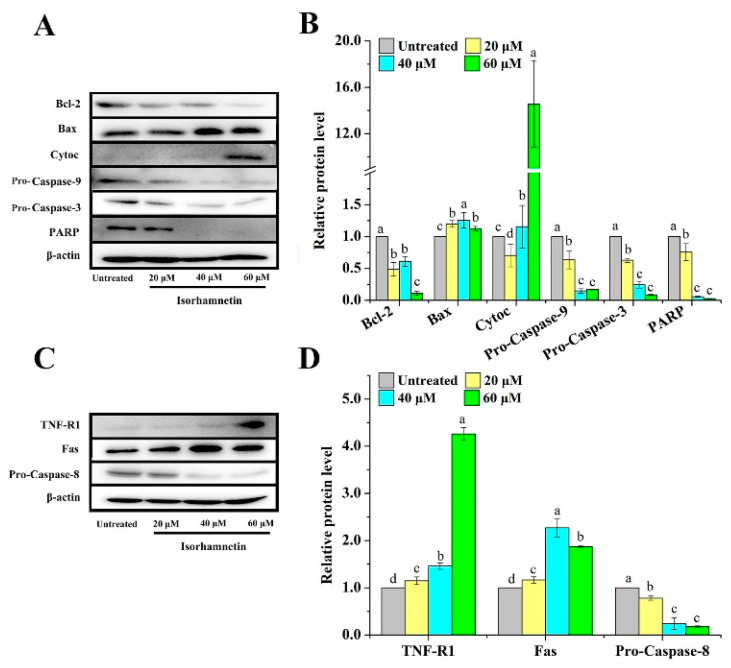
Endogenous and exogenous key proteins of apoptosis for Ishikawa cells treated with different concentrations of isorhamnetin (0 μM, 20 μM, 40 μM, and 60 μM). (**A**): Consequence of isorhamnetin treatment on endogenous apoptosis key proteins Bcl-2, Bax, Cytoc, Pro-Caspase-9, Pro-Caspase-3, and PARP in Ishikawa cells. (**B**): Quantitative protein map of the effect of isorhamnetin on the key proteins of endogenous apoptosis (Bcl-2, Bax, Cytoc, Pro-Caspase-9, Pro-Caspase-3, and PARP). (**C**): Effect of isorhamnetin on the key proteins of exogenous apoptosis (TNF-R1, Fas, and Pro-Caspase-8) in Ishikawa cells. (**D**): Quantitative protein map of the effect of isorhamnetin on the key proteins of exogenous apoptosis (TNF-R1, Fas, and Pro-Caspase-8). Different alphabetic letters (a, b, c, d) denote significant differences between different groups (*p* < 0.05, *n* ≥ 3).

In recent years, various types of programmed cell death have been reported, including the classical apoptotic pathway, autophagy, pyroptosis, ferroptosis, and copper death [38,39,40]. Among them, the apoptotic pathway, as one of the most classical forms of programmed death, is subdivided into the endogenous apoptotic pathway and the exogenous apoptotic pathway [41]. In terms of the endogenous signaling pathway regulating apoptosis, isorhamnetin can inhibit the expression of the apoptosis inhibitor Bcl-2 and conversely promote the expression of the pro-apoptosis protein Bax (Figure 6A,B). The mitochondrial signaling pathway, known as the endogenous signaling pathway, is composed of enzymes and proteins that regulate mitochondria, particularly the Bcl-2 family of mitochondrial enzymes that regulate apoptosis [42]. When the ratio of pro/anti-apoptotic proteins increases, it allows for Cytochrome C to increase, and the precursor Caspase-9 then forms apoptotic vesicles inducing the activation of Pro-Caspase-9 which ultimately leads to the eventual apoptosis of the cell via Caspase-3. In terms of extracellular signaling pathways regulating apoptosis, the death receptor signaling pathway is a pathway that exogenously causes apoptosis in cells and, so far, several pairs of ligand/death receptor signaling systems [43,44,45]. Upon activation of the ligand/death receptor signaling system, the death receptor binding protein (FADD) and Pro-Caspase-8 are recruited to form the death-inducing signaling complex (DISC). After Pro-Caspase-8 activation, Pro-Caspase-3 is sheared and apoptosis is induced.

In addition, endoplasmic reticulum stress is also involved in apoptosis. Importantly, TNF-R1 protein expression was activated, which then triggered the activation of its downstream Caspase-8, resulting in a decrease in the expression of Pro-Caspase-8 and ultimately inducing apoptosis (Figure 6C,D).

### 3.8. Effect of Isorhamnetin on Immunohistochemical Assessment In Vivo

**Figure 7:**
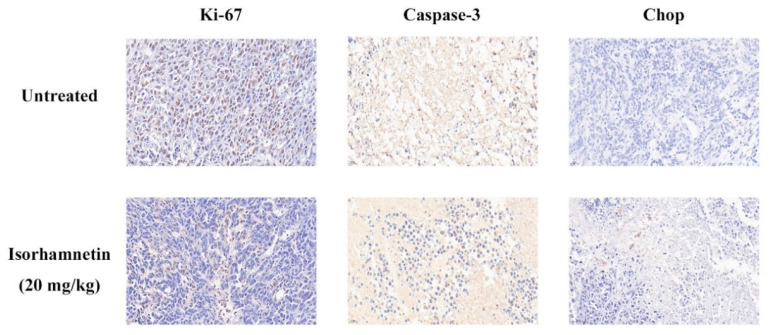
Immunohistochemistry of key proteins Ki-67, Caspase-3, and Chop (the key protein of cell proliferation, key protein of cell apoptosis, and key protein of endoplasmic reticulum stress-induced cell apoptosis, respectively).

Ki-67 is an indicator of cell proliferation in immunohistochemistry, and the higher the value of this indicator, the higher the malignancy of tumor cells [46]. The positive brown areas in the isorhamnetin-treated group were found to be significantly reduced, which means that isorhamnetin is beneficial in alleviating the malignancy of tumor cells. Immunohistochemical staining with Caspase-3 as the key protein of apoptosis showed a significant decrease in the positive brown area in the isorhamnetin-treated group. Chop, as the key protein of endoplasmic reticulum stress, showed a significant increase in the positive brown area in the isorhamnetin-treated group (Figure 7). The above results indicated that isorhamnetin could affect the expression of Caspase-3 (a key protein of apoptosis) and Chop (a key protein of endoplasmic reticulum stress in Ishikawa tumor-bearing mice with endometrial cancer).

### 3.9. Reactive Oxygen Species Levels of Ishikawa Cells Treated by Isorhamnetin

Oxygen radicals generated by ROS are normal products of cellular metabolism and are “double-edged swords” within the cell [47,48,49]. During cellular stress and injury, the cell response is regulated by some levels of superoxide dismutase (SOD), glutathione (GSH), and vitamins through enzymatic and non-enzymatic reactions, and these can reduce the production of ROS [50]. However, once cells experience prolonged oxygen radical invasion, the balance between the rate of oxygen radical production and elimination is disturbed, which in turn affects the normal cell growth process.

**Figure 8:**
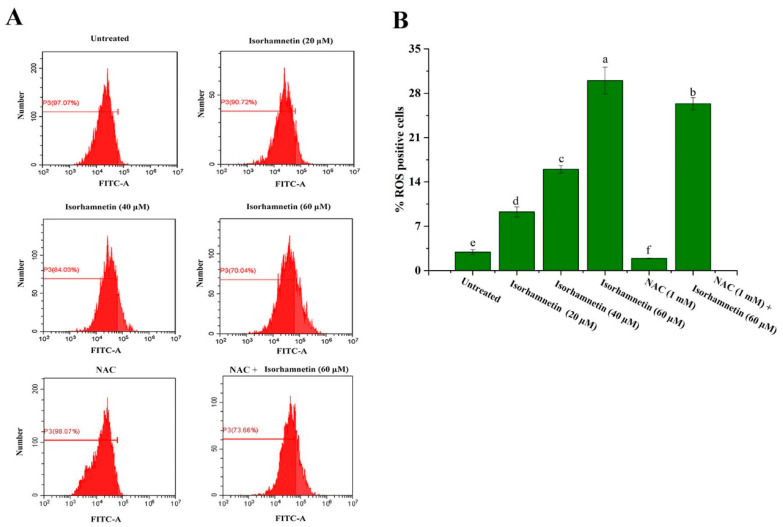
Effect of isorhamnetin on reactive oxygen species production. (**A**): Flow diagram of the effect of isorhamnetin on reactive oxygen species production. (**B**): Quantitative analysis of the effects of isorhamnetin on reactive oxygen species production in Ishikawa cells. Different alphabetic letters (a, b, c, d, e, f) denote significant differences between different groups (*p* < 0.05, *n* ≥ 3).

As shown in Figure 8A,B, the levels of cellular ROS production were 2.93% ± 0.367%, 9.28% ± 0.783%, 16% ± 0.593%, and 30% ± 2.11%, respectively. N-acetylcysteine (NAC) was added in the same way as in previous studies [22]. When NAC was added, the level of cellular ROS production in isorhamnetin was 26.34% ± 0.95%. These above results suggest that isorhamnetin can promote the ROS level.

### 3.10. Effect of Isorhamnetin on Metastasis of Ishikawa Cells and Ishikawa Xenografts in Mice

**Figure 9:**
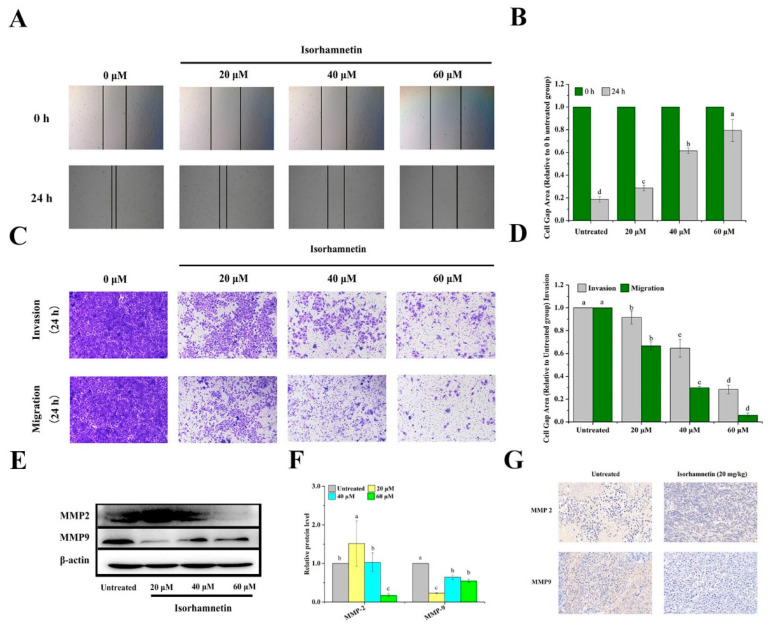
Metastasis of Ishikawa cells and Ishikawa xenografts in mice treated with isorhamnetin. (**A**): Scratch healing of Ishikawa cells with different concentrations of isorhamnetin (0 μM, 20 μM, 40 μM, and 60 μM). (**B**): Quantification of the effects of isorhamnetin (0 μM, 20 μM, 40 μM, and 60 μM) on scratch healing. (**C**): Migration and invasion of Ishikawa cells treated with different concentrations of isorhamnetin (0 μM, 20 μM, 40 μM, and 60 μM). (**D**): Quantification of the migration and invasion of Ishikawa cells treated with different doses of isorhamnetin (0 μM, 20 μM, 40 μM, and 60 μM). (**E**): Effect of isorhamnetin on Ishikawa cell migration and invasion toward MMP2 and MMP9. (**F**): Quantification of the effects of isorhamnetin on MMP2 and MMP9 in terms of Ishikawa cell migration and invasion. (**G**): Immunohistochemistry of key proteins in Ishikawa xenograft mice treated with isorhamnetin (20 mg/kg). Different alphabetic letters (a, b, c, d) denote significant differences between different groups (*p* < 0.05, *n* ≥ 3).

The effects of isorhamnetin on scratch healing in Ishikawa cells are shown in Figure 9A,B. Compared to 0 h, the cell scratches in the control group (0 µM isorhamnetin) were significantly healed after 24 h. However, the rate of cell scratch healing with different concentrations of isorhamnetin was reduced after 24 h treatment. Tumor metastasis is a complex process involving cell migration, invasion, and adhesion, and the ability of cell migration and invasion is usually detected using Transwell assays [51] (Figure 9C,D). MMP2 and MMP9 are metalloprotein kinases which degrade tissue barriers; furthermore, they are also related to epithelial–mesenchymal transition and are associated with cell migration and invasion [52]. At the cellular protein expression level, it can be found that the expression of MMP2 and MMP9, the key metalloprotein kinases associated with migration, was down-regulated in a concentration-dependent manner. All the results express that isorhamnetin can reduce cell migration and invasion in a concentration-dependent manner (Figure 9E,F). Immunohistochemical staining demonstrated that MMP2 and MMP9 are the key proteins of migration and invasion-related pathways [53] (Figure 9G). The establishment of cell adhesion junctions was regulated by E-cadherin, resulting in the formation of a continuous adherent epithelium. If there is an absence of E-cadherin in mesenchymal cells, the tight cell-to-cell junctions are lost. The inhibition of cell migration by isorhamnetin in epithelial–mesenchymal transition deserves further investigation. The results of MMP2 immunohistochemistry showed that the positive brown area after isorhamnetin action was significantly weaker compared to the control group, and MMP9 also showed the same trend.

**Figure 10:**
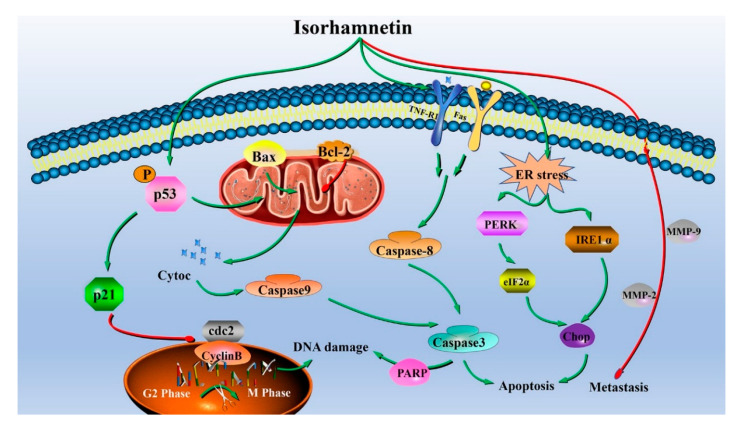
Diagram of the possible molecular mechanism of the anticancer effect of isorhamnetin on human EC Ishikawa cells and Ishikawa xenograft mice.

Altogether, isorhamnetin affected the expression of proteins in the cell cycle, the endoplasmic reticulum stress-related pathway, the mitochondria-related pathway, and the death receptor-related pathway by regulating the intracellular ROS level, and finally induced apoptosis in human EC Ishikawa cells while also inhibiting cell metastasis, as revealed in Figure 10.

## 4. Conclusions

In conclusion, our findings suggest that isorhamnetin induced apoptosis in Ishikawa cells by inducing the endogenous mitochondrial apoptotic pathway and exogenous death receptor pathway while also promoting the endoplasmic reticulum stress-related pathway and activating the corresponding markers of UPR response. In addition, isorhamnetin affected the expression of MMP2 and MMP9-related proteins and eventually inhibited the metastasis of human endometrial cancer Ishikawa cells. In Ishikawa tumor-bearing mice, isorhamnetin inhibited tumor growth through the key proteins of mitochondrial and endoplasmic reticulum-related pathways. Thus, isorhamnetin could be considered a promising natural compound with therapeutic and/or preventive effects against cancer.

## Data Availability

The data used to support the findings of this study can be made available by the corresponding author upon request.
